# The premature abandonment of thoracic epidural analgesia in the Era of video-assisted thoracoscopic surgery thoracic epidural analgesia in the VATS Era

**DOI:** 10.1016/j.bjane.2026.844846

**Published:** 2026-10-10

**Authors:** Gabriel Trindade Avelar, André Versiani Caldeira Rocha, Regina Bicalho Gomes de Faria, Marina Ayres Delgado

**Affiliations:** Universidade Federal de Minas Gerais (UFMG), Hospital das Clínicas, Department of Anesthesiology, Belo Horizonte, MG, Brazil

Dear Editor,

The evolution from open thoracotomy to Video-Assisted Thoracic Surgery (VATS) has significantly modified perioperative analgesia strategies. Contemporary procedure-specific recommendations, most notably the PROSPECT guidelines for VATS, now advocate the thoracic Paravertebral Block (PVB) or Erector Spinae Plane Block (ESPB) as first-choice regional techniques rather than Thoracic Epidural Analgesia (TEA).^1^ These approaches have gained ground because they are less invasive and better aligned with enhanced-recovery pathways. Contemporary evidence therefore supports moving away from routine TEA for most VATS patients; however, whether TEA has no role in any clinical circumstance remains uncertain.^1^

The randomized trials comparing TEA and PVB illustrate this trade-off rather than supporting a uniform conclusion. Lai et al. randomized 86 patients undergoing VATS lobectomy and found lower pain scores at rest and during coughing at 24 and 48 hours with TEA. However, TEA was associated with more vomiting (32.6% vs. 11.6%), dizziness (55.8% vs. 12.9%), pruritus (27.9% vs. 2.3%), and hypotension (32.6% vs. 4.7%).^2^ In contrast, Wu et al., in a randomized trial of 176 patients, found no significant difference in pain scores between PVB and TEA. PVB required less time for catheter placement (median 5 vs. 14 minutes), had a lower catheter-placement failure rate, and was associated with lower incidences of hypotension and urinary retention.^3^ These studies highlight the central trade-off: TEA may provide greater analgesic intensity in some comparisons, whereas PVB may provide similar analgesia with practical and safety advantages in others.

Nevertheless, the safety argument favoring peripheral techniques also deserves appropriate context. Less invasive does not mean risk-free; rather, TEA and peripheral blocks have different technical and adverse-effect profiles that should be weighed alongside analgesic efficacy and recovery outcomes.

The recent multicenter randomized non-inferiority trial provides important contemporary context. Among 450 randomized patients, 389 were included in the intention-to-treat analysis. The proportion of pain assessments with NRS scores ≥ 4 was 20.7% with TEA, 35.5% with PVB, and 29.5% with Intercostal Nerve Block (ICNB). Using a prespecified non-inferiority margin of 17.5%, ICNB met the criterion for non-inferiority to TEA, whereas PVB was inferior to TEA for the primary pain outcome in both intention-to-treat and per-protocol analyses.^4^ Both PVB and ICNB nevertheless reduced opioid consumption and improved mobility compared with TEA, while quality-of-recovery scores were similar across groups.^4^ These findings reinforce that non-inferiority should not be interpreted as equivalence and that analgesic efficacy must be weighed against recovery-related outcomes. Importantly, the trial excluded patients receiving long-term opioid therapy.^4^ This exclusion identifies an evidence gap rather than demonstrating that such patients would benefit more from TEA. Accordingly, patients with chronic opioid use, difficult-to-control pain, or severely compromised respiratory reserve should not yet be considered validated indications for preferential TEA. Instead, they represent clinically relevant populations in whom the comparative effectiveness and net benefit of TEA versus peripheral techniques remain least certain and warrant prospective investigation.

The magnitude of TEA's analgesic advantage should also be interpreted cautiously. A recent systematic review and meta-analysis including four randomized trials and four observational studies (946 patients) found that epidural analgesia reduced pain compared with systemic opioids by a pooled mean difference of 0.8 NRS/VAS points at rest and 1.1 points during activity over postoperative days 1 to 3.^5^ These differences were below or at the lower bound of the commonly cited 1- to 2-point minimal clinically important difference, and heterogeneity was substantial.^5^ Thus, statistically stronger analgesia does not necessarily translate into a clinically meaningful advantage for the average VATS patient.

Taken together, contemporary evidence supports moving away from routine TEA for most patients undergoing VATS. Procedure-specific recommendations favor PVB or ESPB, and peripheral techniques generally offer a favorable balance between analgesia, recovery, and avoidance of neuraxial adverse effects.^1^^,^^4^ At the same time, randomized evidence shows that TEA can provide stronger analgesia in some comparisons, and current trials do not adequately define whether particular higher-risk subgroups derive a clinically meaningful net benefit from TEA.^2^^,^^4^

Therefore, the clinical paradigm must not simply swing from uncritical routine use to indiscriminate complete abandonment.^1^ TEA should no longer serve as the default technique for VATS; instead, its potential role should be considered selectively and on an individual basis when the anticipated analgesic benefit may outweigh its disadvantages.

The appropriate conclusion is therefore not that TEA should remain preferred for a predefined high-risk group, but that its routine use should be abandoned without assuming that its role has been eliminated altogether. Future comparative studies should specifically address patients with chronic opioid use, difficult-to-control pain, and severely compromised respiratory reserve. Until such evidence is available, TEA is best regarded as a non-routine option whose potential value in carefully selected patients remains to be established.

## Data availability statement

No datasets were generated or analyzed for this Letter to the Editor; data sharing is therefore not applicable.

## Declaration of the use of AI and AI-assisted technologies

During the preparation of this manuscript, the authors used ChatGPT (OpenAI) for language editing and improvement of clarity and readability. The authors reviewed and edited all AI-assisted content and take full responsibility for the accuracy, originality, and integrity of the final manuscript. AI and AI-assisted technologies were not used to generate, analyze, or interpret scientific data and select or interpret scientific evidence, nor to make scientific or clinical decisions, and were not used to formulate the clinical conclusions, or generate or verify references.

## Authors’ contributions

Gabriel Trindade Avelar: Conceptualization; investigation; writing-original draft preparation.

André Versiani Caldeira Rocha: Conceptualization; investigation; writing-review & editing.

Regina Bicalho Gomes de Faria: Conceptualization; investigation; writing-review & editing.

Marina Ayres Delgado: Conceptualization; supervision; writing-review & editing; project administration.

## Funding

None.

## Conflicts of interest

The authors declare no conflicts of interest.
